# The Role of Pancreatic Alpha Cells and Endothelial Cells in the Reduction of Oxidative Stress in Pseudoislets

**DOI:** 10.3389/fbioe.2021.729057

**Published:** 2021-09-09

**Authors:** Fredrik C. Wieland, Mireille M.J.P.E. Sthijns, Thomas Geuens, Clemens A. van Blitterswijk, Vanessa L.S. LaPointe

**Affiliations:** ^1^ MERLN Institute for Technology-Inspired Regenerative Medicine, Maastricht University, Maastricht, Netherlands; ^2^ Centre for Healthy Eating and Food Innovation, Maastricht University, Maastricht, Netherlands

**Keywords:** GLP-1 - glucagon-like peptide-1, INS1E cells, HUVEC - human umbilical vein endothelial cell line, oxidative stress, pseudoislet, ROS - reactive oxygen species, alpha TC1 clone 6

## Abstract

Pancreatic beta cells have inadequate levels of antioxidant enzymes, and the damage induced by oxidative stress poses a challenge for their use in a therapy for patients with type 1 diabetes. It is known that the interaction of the pancreatic endocrine cells with support cells can improve their survival and lead to less vulnerability to oxidative stress. Here we investigated alpha (alpha TC-1), beta (INS1E) and endothelial (HUVEC) cells assembled into aggregates known as pseudoislets as a model of the pancreatic islets of Langerhans. We hypothesised that the coculture of alpha, beta and endothelial cells would be protective against oxidative stress. First, we showed that adding endothelial cells decreased the percentage of oxidative stress-positive cells. We then asked if the number of endothelial cells or the size (number of cells) of the pseudoislet could increase the protection against oxidative stress. However, no additional benefit was observed by those changes. On the other hand, we identified a potential supportive effect of the alpha cells in reducing oxidative stress in beta and endothelial cells. We were able to link this to the incretin glucagon-like peptide-1 (GLP-1) by showing that the absence of alpha cells in the pseudoislet caused increased oxidative stress, but the addition of GLP-1 could restore this. Together, these results provide important insights into the roles of alpha and endothelial cells in protecting against oxidative stress.

## Introduction

Diabetes mellitus type 1 is an autoimmune disease that results in the depletion of pancreatic beta (*β*) cells that produce insulin to regulate glucose uptake. The pancreatic tissue is one of the most metabolically active tissues within the human body, and especially the *β* cells at elevated glucose concentrations rely on oxidative metabolism for adenosine triphosphate synthesis (Bakker et al., 2000). To illustrate this, the islets of Langerhans receive up to 10% of the pancreas’ blood supply, but only occupy 1–2% of its volume (Myasnikova et al., 2019).

The result of the high metabolic activity of the pancreatic islets is the generation of by-products such as reactive oxygen species (ROS) by mitochondrial respiration during glucose stimulation (Gerber and Rutter, 2017). An antioxidant defense can be generated by glutathione and vitamin E, as well as with antioxidant enzymes such as superoxide dismutase, catalase, and peroxiredoxins to counteract the formation of ROS. The *β* cells are notably vulnerable to damage induced by oxidative stress as they have inadequate levels of antioxidant enzymes (Haskins et al., 2003; Stancill et al., 2019).

A number of studies suggest an association between endothelial cell incorporation in islets and the cells’ resilience to oxidative stress and improved viability and function (Skrzypek et al., 2018). Endothelial cells are important in establishing the islets of Langerhans’ vasculature (Ballian and Brunicardi, 2007; Berclaz et al., 2016) and they provide extracellular matrix (ECM) components such as type IV collagen and laminins and growth factors that improve *β* cell insulin secretion (Olerud et al., 2011; Figliolini et al., 2014; Komatsu et al., 2017). To understand why supporting cells positively affect endocrine cells, observational studies of their spatial arrangement and interactions have been performed. For example, it has been shown that *β* cells are typically located near endothelial or alpha (*α*) cells (Bosco et al., 2010; Wieland et al., 2020). This localization of *β* cells is associated with functional improvement and can be linked to increased antioxidant protection provided by the *α* or endothelial cells. Therefore, we were interested to understand if the endothelial or *α* cells protect against oxidative stress in islets.

There is also a close functional relationship between *α* and *β* cells that is affected by oxidative stress. Their hormones, insulin and glucagon, counteract each other, where high blood glucose concentration stimulates *β* cells to secrete insulin and suppresses glucagon secretion from the *α* cells (Xu et al., 2006). The *α* cells also secrete an incretin, glucagon-like peptide 1 (GLP-1), upon the stimulus of insulin or high glucose concentration (Liu et al., 2018). High glucose, inflammation and oxidative stress have an effect on GLP-1 production. Normally glucagon is a product of the proglucagon protein cleavage by the enzyme proconvertase 2 (PC2), but stress causes a change in the cleavage of proglucagon by activating the enzyme proconvertase 1/3 (PC1/3), which results in the product GLP-1 (Sancho et al., 2017). In turn, GLP-1 reduces endoplasmic reticulum stress and autophagy in endothelial cells and improves the antioxidant capacity in *β* cells (Ishibashi et al., 2010; Schisano et al., 2012; Fernández-Millán et al., 2016; Cai et al., 2018).

This study set out to assess the roles of *α* and endothelial cells in protecting bioengineered islets against oxidative stress. To do this, we made use of the well-established lines of α (alpha TC-1), β (INS1E) and endothelial (HUVEC) cells that we aggregated in a three-dimensional suspension to form a pseudoislet. This model confers the possibility to control the pseudoislet composition, allowing us to assess the impact of the *α* and endothelial cells on ROS levels, which we did by including or excluding them from the pseudoislet. In the same fashion, we challenged the pseudoislet composition by changing its size (number of cells) or tuning the ratio of endothelial cells. Finally, we investigated the impact of GLP-1 in oxidative stress and showed the important role for *α* cell. The work presented here provides new understanding of the supportive role of *α* and endothelial cells in reducing the oxidative stress in the pseudoislet, which can be taken into account in the future development of bioengineered islets.

## Methods and Material

### Cell Culture

Alpha TC1 clone 6 (Cat# CRL-2934 ATCC, Manassas, United States), referred to as *α* cells, were cultured in DMEM 6046 (Sigma-Aldrich) supplemented with 10% (vol/vol) FBS (Sigma-Aldrich), 15 mM HEPES, 0.1 mM non-essential amino acids, 1.5% (wt/vol) sodium bicarbonate and 2.0% (wt/vol) glucose. INS-1E (AddexBio, San Diego, United States), referred to as *β* cells, were cultured in RPMI 1640 (Gibco) supplemented with 1 mM sodium pyruvate, 0.05 mM 2-mercaptoethanol, 10 mM HEPES, and 5% (vol/vol) FBS. Human umbilical vein endothelial cells (HUVECs) (C2519A, Lonza, Maryland, United States; used at passage 5) were cultivated in EGM-2 (PromoCell). All cells were cultured under a humidified atmosphere with 5% CO_2_ at 37°C and negative for *mycoplasma* contamination (*Mycoplasma* Detection Kit, #B39035, BioTool).

### Creation of Constructs and Stable Cell Lines

The open reading frames of mTagBFP2 and mNeongreen2 were amplified by PCR from donor vectors pBAD-mTagBFP2 (Addgene, #34632) and pSFFV_mNG2 (11)1–10 (Addgene, #82610) respectively, and ligated in a pLenti6.2 destination vector. Both vectors were verified by Sanger sequencing and deposited to Addgene as pLenti6.2_mTagBFP2 (#113725) and pLenti6.2_mNeonGreen2 (#113727).

Stable cell lines were generated using the lentiviral transduction of *α* cells and *β* cells using pLenti6.2_mTagBFP2 and pLenti6.2_mNeonGreen2, respectively. Both plasmids were co-transfected separately with third-generation lentiviral packaging and envelope vectors [pMD2. G (Addgene, #12259), pRSV-Rev (Addgene, #12253), and pMDLg/pRRE (Addgene, #12251)] into HEK-293T cells using the PEIpro (VWR) transfection reagent. After 24 h, the viral supernatant was collected and used to transduce *α* and *β* cells. Positive cells were selected 48 h after transduction using 1 μg/ml puromycin dihydrochloride (Sigma-Aldrich) added to growth media for at least 7 days. Transduction efficiency was assessed by fluorescence microscopy after a minimal culture period of 7 days.

### Stable Cell Line Purification by Using Fluorescence-Activated Cell Sorting

In order to purify the *α*-BFP2 and *β*-mNeonGreen2 cells, a BD FACSAria III equipped with a 100 μm flow tip facilitated the sorting of the cells. Untransfected cells were used as an autofluorescence control. The cells were sorted in PBS supplemented with 10% (vol/vol) FBS (Sigma-Aldrich). Debris was excluded by gating on the physical parameter (FSC-A/SSC), followed by excluding doublets using pulse processing (FSC-H vs FSC-A). Sorted cells were collected in a 15 ml tube with 3 ml of culture medium.

### Pseudoislet Formation

Prior to cell seeding, the wells were washed twice with modified EGM-2 medium supplemented with a final concentration of 10 mM HEPES, 1 mM sodium pyruvate, 0.1 mM non-essential amino acids, 2% (wt/vol) glucose and 0.05 mM 2-mercaptoethanol. Cell number was determined by trypan blue exclusion on an automated cell counter (TC20, Bio-Rad Laboratories). In order to form pseudoislets, which are three-dimensional aggregates of *α*, β and endothelial cells, the cells were trypsinised with 0.05% trypsin-EDTA for 5 min at 37°C. Subsequently, the cells seeded either in a 24-well AggreWell 400 plate (STEMCELL Technologies) to generate 1,200 pseudoislets/well or in a 96-well round-bottom plate (Corning Elplasia) for 79 pseudoislets/well. As a basis, all pseudoislets had a total of 1,500 cells, but their composition had the varying ratios of *α*:*β* (1:14), *α*:*β*:EC (1:9:5), 1× *α*:*β*:EC (1:9:2), 2× *α*:*β*:EC (1:9:5) and 4× *α*:*β*:EC (1:9:20). When studying the different sizes of pseudoislets, the ratio of *α*:*β*:EC of 1:9:5 was used with the different total number of 750, 1,500 and 3,000 cells. All cell types were seeded simultaneously into the microwell array, which was then centrifuged at 200 × g for 4 min to distribute the seeded cells into the microwells evenly. The pseudoislets were cultured for up to 5 days in a modified EGM-2 medium, with the medium refreshed daily.

### ROS Assay

To induce oxidative stress, the pseudoislets were incubated with 250 µM hydrogen peroxide (H_2_O_2_) diluted in phenol-free EGM-2 (PromoCell) after being washed with PBS for 2 min. After 30 min incubation in H_2_O_2,_ 10 µM of the fluorogenic probe CellROX Deep Red Reagent (Invitrogen) in phenol-free EGM-2 with/without 250 µM H_2_O_2_, was added into the existing medium to a final concentration of 5 µM. Control samples were incubated with phenol-free EGM-2 for 60 min at 37°C. After 30 min of incubation with CellROX Deep Red Reagent, the pseudoislets were fixated in 4% (wt/vol) formaldehyde diluted in PBS for 20 min at room temperature and were subsequently, washed two times with PBS for 3 min.

### Glucagon Like-Peptide 1

The glucagon-like peptide-1 (GLP-1) agonist, liraglutide (Abcam), was prepared in the modified EGM-2 at a final concentration of 100 nM. It was pre-incubated with the pseudoislet for 2 h under a humidified atmosphere with 5% CO_2_ at 37°C. In the ROS assay, the condition with GLP-1 pre-incubation had 100 nM GLP-1 agonist included in the phenol-free EGM-2 media with 250 µM H_2_O_2_.

### Analysis to Identify ROS Positive Cells

The pseudoislets were incubated with 5 µM SYTOX Orange nucleic acid stain (Invitrogen) diluted in PBS for 30 min followed by two washes with PBS. They were gently flushed out of the microwell arrays into the CELLview dish (Greiner Bio-One) and mounted with ProLong Gold (Invitrogen). To identify ROS-positive cells, optical sections (z-stacks; 60–90 µm into the depth of the pseudoislet) were obtained on a Nikon Eclipse Ti-E inverted microscope equipped with a 40×/1.3 NA immersive oil objective (Nikon Instruments) and spinning disc X-Light2 (CrestOptics).

Cell identification and quantification was done using Imaris 9.7.0 (Bitplane, South Windsor, CT, United States). Images were automatically reconstructed into 3D visualisations and the “spot” tool identified the nuclei using SYTOX Orange with an estimated diameter set to 6 µm and a manual threshold setting. To quantify *α* cells with BFP2 (Ex *λ* 399 nm and Em *λ* 454 nm), the “surface” tool was applied followed with the parameters: 12 µm diameter of largest sphere, 10 µm split touching objects, and a manual threshold setting. To quantify *β* cells with mNeonGreen2 (Ex *λ* 506 nm and Em *λ* 517 nm), the “surface” tool was applied followed with the parameters: 16 µm diameter of largest sphere, 8 µm split touching objects, and a manual threshold setting. To identify ROS-positive cells with CellROX Deep Red (Ex *λ* 644 nm and Em *λ* 665 nm), the “surface” tool was applied with a threshold set using the control samples. To quantify the number of positive nuclei with oxidative stress, the function “shortest distance to surfaces” was used to identify a nucleus within 5 µm distance (in the planes of xyz) to ROS. To count ROS-positive *α* and *β* cells, the function “shortest distance to surfaces” was used and cells were considered positive when the distance of the cells’ surface was within 1 µm to the ROS dye. Any manual threshold settings were kept constant in all experiments.

### Statistical Analysis

Data were reported as means ± SEM from three to four independent experiments (N = 3–4), each of which included a set of 10–16 pseudoislets (n = 10–16). Statistical analysis was done in Prism software (GraphPad, version 8.1, La Jolla, CA, United States), with significance determined by a 2-way ANOVA with Holm-Šidák’s multiple comparison test when *p* ≤ 0.05.

## Results

### The Addition of Endothelial Cells in the Pseudoislet Reduces the Oxidative Stress in *α* and *β* Cells

**FIGURE 1:**
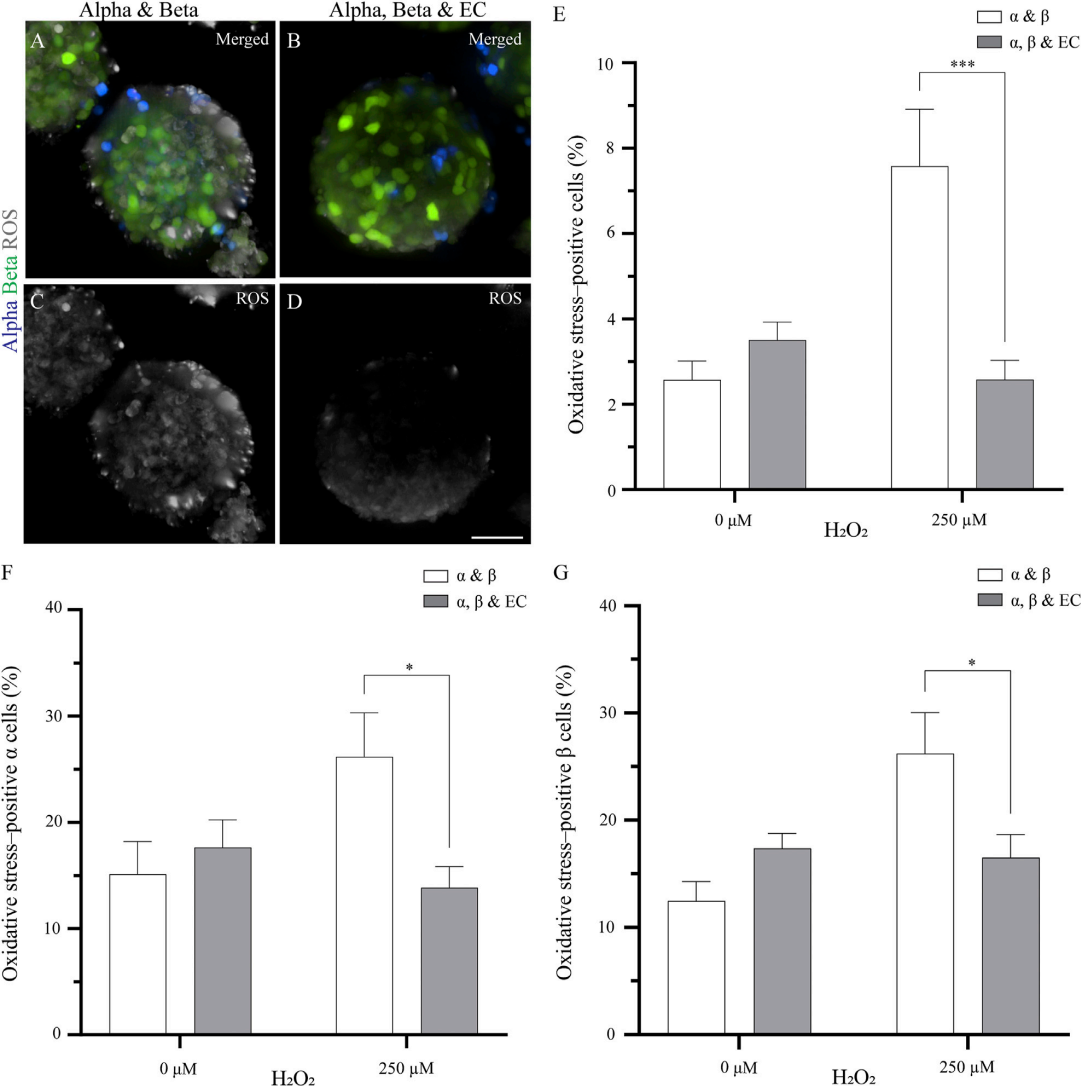
The addition of endothelial cells in the pseudoislet reduces the oxidative stress in *α* and *β* cells **(A, B)** Fluorescence imaging reveals ROS-positive cells in samples that were exposed to 250 µM H_2_O_2_, showing *α* cells (blue), *β* cells (green) and ROS (grey) **(C, D)** Oxidative stress varies in pseudoislets with and without endothelial cells. A lower percentage of oxidative stress-positive cells are found in the pseudoislets including endothelial cells. Scale bars: 50 µm **(E)** After 5 days in culture, the endothelial cells decrease oxidative stress (***p < 0.001) **(F)** The presence of endothelial cells in the pseudoislet did not affect the percentage of oxidative stress-positive *α* cells in the basal media, however when induced by H_2_O_2_, a significant decrease of oxidative stress-positive *α* cells was seen (*p < 0.04) **(G)** The inclusion of endothelial cells in the pseudoislet reduced the percentage of oxidative stress-positive *β* cells upon induction by H_2_O_2_ (*p < 0.04). Results are expressed as mean ± SEM, and each data set includes twelve pseudoislets (n:12), and the experiment was repeated three times (N:3).

In order to describe the role that endothelial cells play in the response of pseudoislets to oxidative stress induced by H_2_O_2_, we formed two types of pseudoislets. The first comprising 7% *α* cells and 93% *β* cells and the second comprising 7% *α* cells, 60% *β* cells, and 33% endothelial cells. All pseudoislets contained approximately 1,500 cells. After 5 days in culture, oxidative stress was induced by the addition of H_2_O_2_ for 1 h, after which the cells were labeled with a fluorescent probe (CellROX Deep Red Reagent) for detection of oxidative stress (Figures 1A–D). Overall, the addition of endothelial cells to the pseudoislet had a protective effect against oxidative stress.

By adding endothelial cells to the pseudoislet, the percentage of oxidative stress-positive cells incubated with H_2_O_2_ was not significantly higher than the control condition without H_2_O_2_ (Figure 1E). When the pseudoislets were exposed to 250 μM H_2_O_2_, the presence of endothelial cells reduced the total percentage of cells positive for oxidative stress from 7.6 to 3.6% (*p* < 0.001; Figure 1E; N:3, n:12).

We then wanted to determine how *α* and *β* cells responded to the induction of oxidative stress and how this was affected by including endothelial cells. Looking at the *α* cells, we found that 15.1–17.6% were already positive for oxidative stress in the basal condition without H_2_O_2,_ and this was unaffected by the presence of endothelial cells (Figure 1F; N:3, n:12). However, when oxidative stress was induced by H_2_O_2_, the presence of endothelial cells impacted how the *α* cells responded. Without endothelial cells in the pseudoislet, H_2_O_2_ increased the percentage of oxidative stress-positive *α* cells to 26.2%, but with endothelial cells in the pseudoislet, this was reduced to 13.8% (*p* < 0.04; Figure 1F; N:3, n:12). The *β* cells had comparable percentages of oxidative stress-positive cells in the basal condition without H_2_O_2_ regardless of the presence of endothelial cells (17.4% with vs 12.5% without endothelial cells; Figure 1G; N:3, n:12). However, when oxidative stress was induced by H_2_O_2_, the presence of endothelial cells reduced the percentage of oxidative stress-positive *β* cells from 26.2 to 16.5% (Figure 1G; *p* < 0.04; N:3, n:12).

### The Pseudoislet Size does not Affect the Percentage of Oxidative Stress-Positive Cells

Having determined that endothelial cells in the pseudoislet protected both *α* and *β* cells from oxidative stress induced by H_2_O_2_, we then asked whether the size of the pseudoislet could play a role, as our previous research showed that more endothelial cells were incorporated into the pseudoislet when the size increased (Wieland et al., 2020). To study this, we generated three differently sized pseudoislets with 750, 1,500 or 3,000 cells, all with the composition of 6.7% *α* cells, 60% *β* cells and 33.3% endothelial cells.

**FIGURE 2:**
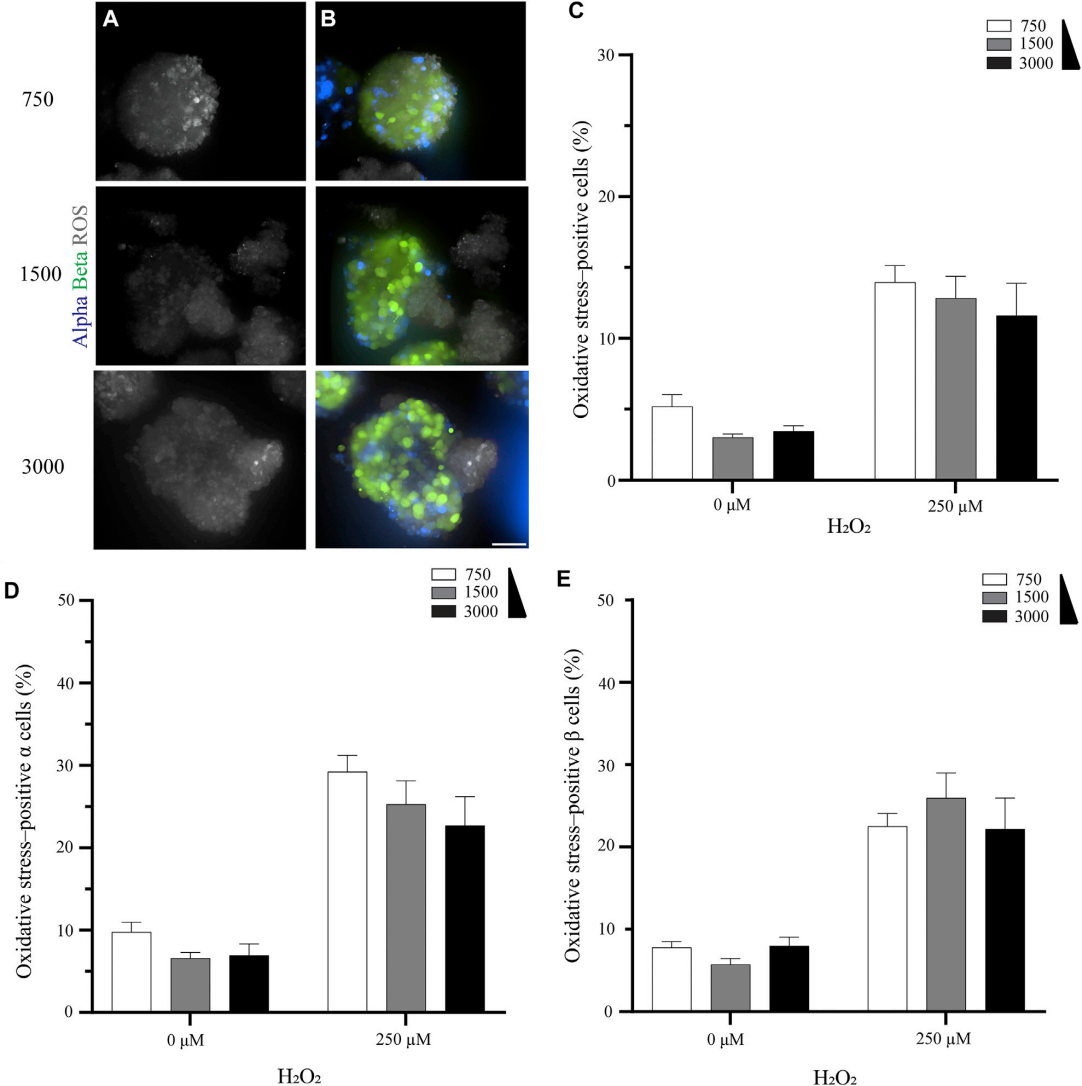
The pseudoislet size does not affect the percentage of oxidative stress-positive cells **(A)** Fluorescence imaging of the different sized pseudoislets (750, 1,500 and 3,000 cells) and the oxidative stress-positive cells in pseudoislets exposed to 250 µM H_2_O_2_ **(B)** The different sized pseudoislets showing *α* cells (blue), *β* (green) and ROS (grey). Scale bars: 50 µm **(C)** The percentage of oxidative stress-positive cells in differently sized pseudoislets was not affected by the induction of oxidative stress with H_2_O_2_ **(D)** The percentage of oxidative stress-positive *α* cells was 22.7–29.2% **(E)** The percentage of oxidative stress-positive *β* cells was 22.2–26.0%. Results are expressed as mean ± SEM, and each data set includes fifteen pseudoislets (n:15), and the experiment was repeated three times (N:3).

Overall, the size of the pseudoislet had no apparent effect on the oxidative stress (Figures 2A,B). This result was confirmed when we quantified the percentage of oxidative stress-positive cells. While the percentage increased when oxidative stress was induced by H_2_O_2_ (from 3.0–5.2% to 11.6–14.0%), as would be expected, it was unaffected by the size of the pseudoislet (Figure 2C). No significant difference was measured between the pseudoislets comprising 750, 1,500, or 3,000 cells in terms of the percentage of oxidative stress-positive *α* cells (22.7–29.2%, *p* > 0.18) or *β* cells (22.2–26.0%, *p* > 0.72) (Figures 2D,E; N:3, n:15).

### The Number of Endothelial Cells does Affect the Percentage of Oxidative Stress-Positive Cells

**FIGURE 3:**
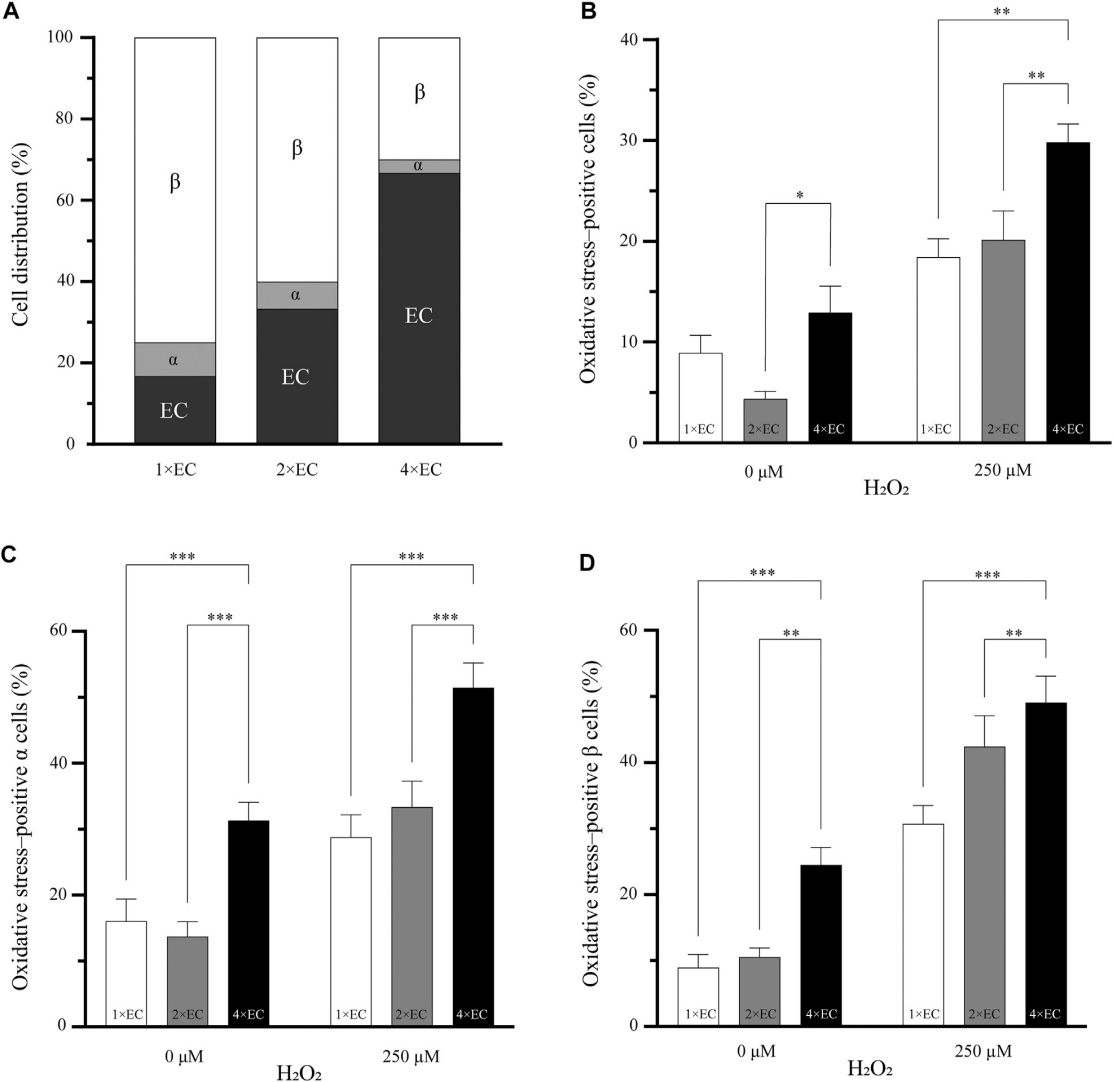
Increasing the number of endothelial cells does decrease the percentage of oxidative stress–positive cells **(A)** Three different seeding conditions of endothelial cells were used to understand if the number of endothelial cells can influence the oxidative stress in the pseudoislet. The cell ratio between α and *β* remained the same in all three conditions **(B)** The two pseudoislets (1× and 2×) with the fewest endothelial cells had a significantly lower number of oxidative stress-positive cells than the 4× pseudoislet with the induction of oxidative stress (*p < 0.03, **p < 0.008) **(C, D)** Looking at the *α* and *β* cells, there were significantly more oxidative stress-positive cells (**p < 0.01, ***p < 0.001) in the 4× pseudoislets. Results are expressed as mean ± SEM, and each data set includes twelve pseudoislets (n:12), and the experiment was repeated three times (N:3).

Our observation that endothelial cells reduced the oxidative stress experienced by the *α* and *β* cells (Figure 1) prompted us to consider whether increasing the quantity of the endothelial cells could further enhance this effect. We began by generating pseudoislets with three different seeding ratios of endothelial cells: 17% (1×), 33% (2×) and 67% (4×). The total number of cells (1,500 cells per pseudoislet) and the ratio between *α* and *β* cells (1:9, respectively) remained constant (Figure 3A).

Figure 3B shows the percentage of oxidative stress-positive cells in the pseudoislets with the different endothelial cell ratios of 1×, 2× and 4×. In the basal condition (without oxidative stress induced by H_2_O_2_), the 2× pseudoislet had significantly lower oxidative stress compared to the 4× pseudoislet (4.3% compared to 12.9%; *p* < 0.03). With the induction of H_2_O_2_, the 4× pseudoislet had significantly more cells positive for oxidative stress than the 1× and 2× pseudoislets (29.8% compared to 18.4 and 20.1%, respectively; *p* < 0.008) (Figure 3B; N:3, n:12).

When looking at the percentage of oxidative stress-positive *α* (Figure 3C) and *β* (Figure 3D) cells, there was also a significant difference between the 4× pseudoislet compared to the 1× and 2× pseudoislets. The lowest percentage of oxidative stress-positive *α* cells was found in the 1× (*p* < 0.001) and 2× (*p* < 0.001) pseudoislets, which was both in the basal condition and with induction of oxidative stress. The 1× pseudoislet had 28.7% oxidative stress–positive α cells, and the 2× pseudoislet had 33.4% oxidative stress-positive *α* cells, which was significantly lower than the 4× pseudoislet that had 51.4% oxidative stress-positive *α* cells upon induction by H_2_O_2_ (Figure 3C; N:3, n:12).

The lowest percentage of oxidative stress-positive *β* cells was also in the 1× and 2× pseudoislets. The 1× pseudoislet had only 8.9% oxidative stress–positive *β* cells in the basal condition without H_2_O_2,_ and with the induction of H_2_O_2,_ this increased to 30.7%. Both conditions had a significantly lower number of oxidative stress-positive *β* cell than the 4× pseudoislet (Figure 3D; *p* < 0.001; N:3, n:12). Similar findings were also observed in the 2× pseudoislet, where the percentage of oxidative stress-positive *β* cells was significantly lower both with or without H_2_O_2_ compared to the 4× pseudoislet (Figure 3D; *p* < 0.01; N:3, n:12).

In summary, we found that the number of endothelial cells in the pseudoislet affected the cells’ oxidative stress. To our surprise, it was not that more endothelial cells enhanced the effect of reducing oxidative stress-positive cells. Instead, we saw that fewer endothelial cells (1× pseudoislet) resulted in the fewest oxidative stress-positive cells. These results suggested that the composition of the pseudoislet may be the leading factor in reducing oxidative stress. Since the 1× pseudoislet had more *α* cells compared to the 4× pseudoislet, we went on to investigate the role of *α* cells in reducing oxidative stress.

### The Incretin GLP-1 that is Secreted by the *α* Cells Reduce Oxidative Stress

Given these findings, we were interested to know whether the *α* cells played a role in protecting against oxidative stress in the pseudoislet. Until this point, we had kept the ratio between the *α* and *β* cells constant while modulating the prevalence of endothelial cells and overall pseudoislet size. Now we created two different conditions of pseudoislets: one with 6.7% *α* cells, 60% *β* cells and 33.3% endothelial cells (ABEC), and one with 0% *α* cells, 66.6% *β* cells and 33.3% endothelial cells (BEC).

**FIGURE 4:**
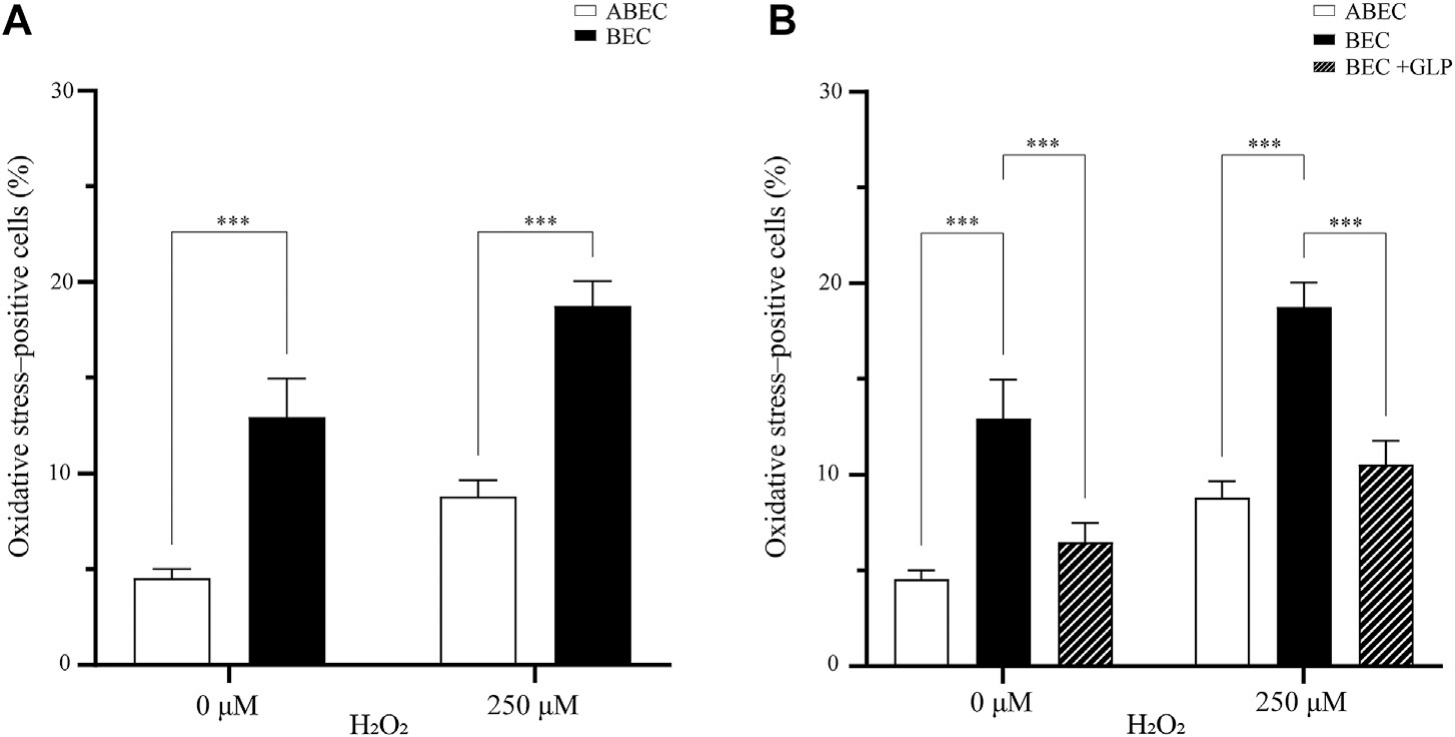
GLP-1 reduces oxidative stress in the pseudoislet **(A)** Including *α* cells in the pseudoislet reduces the percentage of oxidative stress-positive cells from 13.0 to 4.5% in basal conditions and from 18.8 to 8.8% upon induction of oxidative stress by H_2_O_2_ (***p < 0.001) **(B)** The GLP-1 agonist mimicked the effect of having *α* cells present in the pseudoislet devoid of *α* cells, as it reduced the number of oxidative stress-positive cells from 18.8 to 10.5% (***p < 0.001). Results are expressed as mean ± SEM, and each data set includes sixteen pseudoislets (n:16), and the experiment was repeated four times (N:4).

Overall, the presence of *α* cells strongly impacted the oxidative stress in the pseudoislet. In the basal condition (without H_2_O_2_), adding *α* cells reduced the percentage of cells positive for oxidative stress from 13.0 to 4.5% (*p* < 0.001; Figure 4A; N:4, n:16). When H_2_O_2_ induced oxidative stress, this effect was even more pronounced, and the presence of *α* cells reduced the percentage of cells positive for oxidative stress from 18.8 to 8.8% (*p* < 0.001).

To seek a biological basis of this effect, we looked at the glucagon like-peptide 1 (GLP-1), which is secreted by the *α* cells and has shown antioxidative properties. We wondered if a GLP-1 agonist could mimic the presence of *α* cells in the pseudoislet devoid of them. To test this hypothesis, we pre-incubated the pseudoislets with the GLP-1 agonist prior to induction of oxidative stress by H_2_O_2_. Overall, we found evidence that the GLP-1 agonist could replicate the effects of including the *α* cells in the pseudoislet. Without the GLP-1 agonist, the percentage of oxidative stress*-*positive cells induced by H_2_O_2_ increased from 13.0 to 18.8% in the pseudoislets without *α* cells. However, with the GLP-1 agonist, this increase was reduced (from 18.8 to 10.5%), reaching levels statistically similar to those found in the pseudoislets with *α* cells (8.8%; *p* < 0.001; Figure 4B). Together, these results provide important insights into intercellular redox regulation by the composition of the pseudoislets.

## Discussion

The present study was designed to determine how the different cells in the pseudoislet have a supportive role in protecting against oxidative stress. To do this, we used three different cell lines, α (alpha TC-1), β (INS1E) and endothelial (HUVEC) cells that we aggregated together in a three-dimensional suspension to form a pseudoislet. Our current pseudoislet model is limited by the reliance on only *α* cells and *β* cells to represent the islet of Langerhans. This choice was considered carefully as this composition can match the functional level of a primary islet, and in addition, these cell lines are well-characterised (Kim et al., 2015; Skrzypek et al., 2018). Furthermore, the addition of endothelial cells confers complexity to the pseudoislet. It would nonetheless be interesting to consider including *δ* cells (making up ∼10% of the cell population in the islet) in future studies, as they are also known to interact with both *α* and *β* cells (Brereton et al., 2015). These pseudoislets were cultured in microwells for up to 5 days. To induce oxidative stress, we exposed the pseudoislet to H_2_O_2_ for 1 h and subsequently detected the generated ROS with the CellROX Deep Red reagent before imaging with a fluorescence microscope. With this approach, we demonstrated that endothelial cells and *α* cells play an essential role in protecting the pseudoislet against oxidative stress.

Our interest began with the role of the endothelial cells, a cell type that is not only important in establishing a vasculature but also supports other cell types by synthesizing ECM proteins and secreting soluble factors (Cao and Wang, 2014). The addition of endothelial cells to *β* cell cultures is known to increase their viability, insulin secretion, and oxidative stress protection (Brissova et al., 2006; Lau et al., 2012). There is also a link between endothelial cell dysfunction and oxidative stress in patients with type 1 or 2 diabetes stress, where an increase in oxidative stress in the endocrine cells has been reported (Hadi and Suwaidi, 2007; De Mattia et al., 2008). We showed that including endothelial cells in the pseudoislet significantly decreased the percentage of oxidative stress-positive cells from 7.6 to 3.6% upon induction of oxidative stress, a change of 32.1% (Figure 1E).

Looking at which cell types were affected by the inclusion of endothelial cells, we found that their addition to the coculture reduced the percentage of oxidative stress-positive *α* and *β* cells by 34.5 and 38.6%, respectively (Figures 1F,G). This protection is potentially consequential for *β* cells, which are inadequate in antioxidant enzymes and are dysfunctional at high oxidative stress levels (Kaneto et al., 1999; Haskins et al., 2003; Stancill et al., 2019). The reduction of oxidative stress-positive cells can be attributed to the efficient antioxidative effect of the endothelial cells where various enzymes participate in protecting the endothelial cells against oxidative stress, e.g. superoxide dismutase (SOD), thioredoxin, catalase and glutathione peroxidase (Incalza et al., 2018; Sena et al., 2018; Alhayaza et al., 2020).

As far as improving the protective effect of endothelial cells against oxidative stress, many non-enzymatic compounds, such as vitamin C, d-α-tocopherol (vitamin E), glutathione and α-lipoic acid, have been supplemented to cells in *in vitro* and *in vivo* studies with a conclusive reduction in oxidative stress (Matough et al., 2012; Myasnikova et al., 2019). In our approach, we relied on the self-assembly process that forms the pseudoislet. Therefore, we challenged the pseudoislet by changing its size (total number of cells) and the ratio of endothelial cells to understand how they affected oxidative stress protection. Our results showed no significant benefit of changing the size of the pseudoislets from 750 to 3,000 cells per pseudoislet, as the number of oxidative stress-positive cells remained around 12.8% (Figure 2C). This was surprising, as previous studies have shown that larger pseudoislets incorporate more endothelial cells (Bosco et al., 2010; Wieland et al., 2020), suggesting that the protection conferred by the endothelial cells in our study was not directly correlated to their number.

We then examined how changing the ratio of the different cell types in the pseudoislet would affect oxidative stress levels. We focused on changing the prevalence of endothelial cells in the pseudoislet, which could explain if increasing their incorporation had a beneficial effect against oxidative stress, which was previously thought. Interestingly, despite the apparent protective effect of endothelial cells in the coculture, it was not that more endothelial cells gave a greater effect (Figure 3B). This was in agreement with our observation that the number of endothelial cells did not reduce oxidative stress. One possible explanation for this is that the *β* and endothelial cells benefitted from an increased number of *α* cells in the condition with the fewest endothelial cells (1×; Figure 3B). Our observation that both *α* and *β* cells had the fewest cells positive for oxidative stress (28.7 and 30.7%, respectively) in the condition with the most *α* cells (1×, Figures 3C,D) supported this explanation.

For an explanation of why the *α* cells might be protecting the *β* and endothelial cells against oxidative stress, we looked at the incretin hormone GLP-1, which increases cellular levels of antioxidative enzymes, such as glutathione peroxidase and -reductase in *β* cells, and inhibits oxidative stress in endothelial cells (Marchetti et al., 2012; Schisano et al., 2012; Fernández-Millán et al., 2016; Cai et al., 2018). GLP-1 was first found in the enteroendocrine L cells in the early 1980s. It is secreted at a low concentration in the fasting state but rapidly increases after food ingestion to affect blood glucose levels (Drucker, 2018). Previous studies had noted that *α* cells could secrete GLP-1 when metabolically stressed. The posttranslational processing of proglucagon regulates the formation of GLP-1 by the prohormone convertase 1/3. Under normal (low stress) conditions, prohormone convertase 1/3 is inactivated, and prohormone convertase 2 is active in the *α* cells, which generates the hormone glucagon (Sancho et al., 2017; Sandoval, 2020). This is the mechanism by which *α* cells play an essential role in providing antioxidant protection and controlling the glucose level.

In this study, we found that the inclusion or exclusion of *α* cells in the pseudoislet made a significant difference in the percentage of oxidative stress-positive cells. The pseudoislets with *α* cells had 4.5% oxidative stress-positive cells, while those without *α* cells had 13.0% (Figure 4A). We then wanted to determine whether this result may be explained by the fact that GLP-1 is essential for providing antioxidant protection. We were interested to learn that pseudoislets without *α* cells could be pre-incubated with the GLP-1 agonist, liraglutide, and that the percentage of oxidative stress-positive cells was statistically similar to having including the *α* cells (Figure 4B). This suggested that the protective effect of the *α* cells against the induction of oxidative stress could be attributed to their expression of GLP-1.

The underlying mechanism of how GLP-1 (or its agonist) protects against oxidative stress has been thoroughly described. Upon activation of the GLP-1 receptor, the protein kinase C pathway increases glutathione peroxidase and reductase in the *β* cells and inhibits ROS-induced apoptosis by the associated activation of the PI3K/AKT signalling pathway (Buteau et al., 2006; Fernández-Millán et al., 2016). The GLP-1 is also valuable for the endothelial cells as it can inhibit NADPH oxidase, which is elevated in oxidative stress (Oeseburg et al., 2010; Wang et al., 2013). Furthermore, GLP-1 enhanced eNOS and NO expression in endothelial cells to better protect against oxidative stress (Lim et al., 2017). Together, these data substantiate the importance of the support cells in the pseudoislet and explain the characteristic cell organization of the endocrine cells that we previously described (Wieland et al., 2020).

The results reported here shed new light on the beneficial effects of *α* and endothelial cells in protecting against oxidative stress in a three-dimensional coculture with *β* cells. These findings can be applied in the setting of biomaterials for *β* cell or islet encapsulation, where a GLP-1 agonist could be included to protect the cells from the stress induced by the material (Sthijns et al., 2021). The study also has implications for the aim of developing a cell-based therapy of *β* cells from differentiated pluripotent stem cells and suggests that including both *α* and endothelial cells can be beneficial for successful transplantation into patients with type 1 diabetes (Orlando et al., 2014; Sthijns et al., 2018; Augsornworawat et al., 2019; Velazco-Cruz et al., 2019; Yoshihara et al., 2020).

## Data Availability

The datasets presented in this study can be found at: https://doi.org/10.34894/F03MPH.
